# The pivotal role of TGF-β/Smad pathway in fibrosis pathogenesis and treatment

**DOI:** 10.3389/fonc.2025.1649179

**Published:** 2025-09-03

**Authors:** Feilong Chen, Lei Lyu, Chengyuan Xing, Yu Chen, Shaofan Hu, Meng Wang, Zhengdong Ai

**Affiliations:** ^1^ Sports Medicine Key Laboratory of Sichuan Province, Expert Centre of Sichuan Province, Institute of Sports Medicine and Health, Chengdu Sport University, Chengdu, China; ^2^ Jinfeng Laboratory, Chongqing, China; ^3^ Department of Pathophysiology, College of High Altitude Military Medicine, Third Military Medical University (Army Medical University), Chongqing, China; ^4^ Institute for Cancer Medicine, School of Basic Medical Sciences, Southwest Medical University, Luzhou, Sichuan, China

**Keywords:** fibrosis, TGF-β/Smad signalling pathway, ECM, miRNA, EMT

## Abstract

Fibrosis, which is characterized by pathological extracellular matrix (ECM) accumulation impairing organ function, is governed primarily by dysregulated transforming growth factor-β (TGF-β)/Smad signalling. TGF-β1 triggers canonical (Smad2/3-dependent) and noncanonical pathways upon receptor binding, driving profibrotic processes such as fibroblast activation, epithelial–mesenchymal transition (EMT), excessive ECM production (e.g., collagen), and the suppression of matrix degradation. This pathway is central to organ-specific fibrogenesis: In liver fibrosis, it activates hepatic stellate cells (HSCs); in renal fibrosis, it promotes tubular injury and ECM deposition; in pulmonary fibrosis, it induces EMT/fibroblast transition in radiation/bleomycin models; in cardiac fibrosis, it mediates fibroblast activation in diabetic cardiomyopathy/atrial fibrillation via NPRC/TGIF1/USP mechanisms; and in skin fibrosis (e.g., scleroderma), it stimulates collagen overproduction, which is suppressed by osthole or mesenchymal stem cells. The TGF-β/Smad axis thus represents a pivotal therapeutic target. Future research should clarify tissue-specific regulatory networks and develop combinatorial antifibrotic strategies.

## 1 Introduction

Fibrosis is a pathological process characterized by persistent deposition of cells and mediators, leading to structural damage to and dysfunction of normal tissues and organs and significantly impacting patients’ physical and mental health and quality of life (1). Notably, the TGF-β/Smad signalling pathway plays a crucial role in cell biology and disease progression, primarily involving processes such as cell proliferation, growth, differentiation, apoptosis, and ECM regulation (2–4). In recent years, with increasing research, it has become apparent that the TGF-β/Smad pathway plays a pivotal role in the initiation and development of numerous diseases. Its involvement in fibrotic diseases, in particular, has garnered significant attention. Fibrosis in various organs, such as the lung (5), heart (6), kidney (7), and liver (8), is closely associated with aberrant activation of the TGF-β/Smad signalling pathway. For example, in diabetic cardiomyopathy (DCM) models, deficiency of natriuretic peptide receptor C (NPRC) alleviates cardiac fibrosis in diabetic mice by inhibiting the TGF-β/Smad pathway (6). Conversely, in pulmonary fibrosis, selpercatinib inhibits fibroblast proliferation, migration, activation, and ECM deposition by suppressing the TGF-β/Smad pathway, thereby attenuating the progression of lung fibrosis (9).

In this review, we summarize research advances concerning the TGF-β1/Smad signalling pathway, focusing on its role and associated mechanisms in various fibrotic diseases. Building upon this foundation, we analyse the commonalities and differences in the actions of TGF-β across different fibrotic diseases on the basis of the latest research findings and discuss current challenges and future research directions. In subsequent sections, we detail various molecules and regulatory mechanisms related to the TGF-β signalling pathway and analyse their potential roles in disease progression and therapy. In this review, we focus primarily on TGF-β1 and Smad2/3.

## 2 Current research status of TGF-β1

### 2.1 Classification of TGF-β family proteins

The TGF-β family comprises three principal isoforms (TGF-β1, -β2, and -β3) that function as pleiotropic cytokines (10, 11). Upon binding to specific cell surface receptors, TGF-β initiates distinct intracellular signalling cascades, which are classically categorized into Smad-dependent (canonical) and Smad-independent (noncanonical) pathways, with their activation and propagation being precisely regulated through multiple molecular mechanisms (12). The TGF-β receptor system consists of two transmembrane serine/threonine kinase receptors, type I (TβRI) and type II (TβRII) (13). The signalling cascade is initiated by ligand binding to TβRII, which subsequently recruits and phosphorylates TβRI to form an active receptor complex, thereby stimulating its kinase activity to propagate downstream signalling. Importantly, coreceptors, including endoglin (CD105) and betaglycan (TGFβR3), serve as critical modulators of TGF-β signal transduction through their regulatory functions in receptor complex assembly and signal amplification (14).

### 2.2 TGF-β1 biogenesis, activation and signal transduction

The generation, activation, and signal transduction of TGF-β1 constitute a sophisticated biological process. Initially, synthesized in the endoplasmic reticulum as a propeptide, TGF-β1 consists of an N-terminal latency-associated peptide (LAP) and a C-terminal mature TGF-β1 domain. Following biosynthesis, two propeptide monomers form homodimers via disulfide bonds before being transported to the Golgi apparatus (15). Within the Golgi, furin-mediated proteolytic cleavage separates the mature TGF-β1 domain from LAP, although these cleavage products remain noncovalently associated (16, 17). This results in the formation of the small latent complex (SLC), also called latent TGF-β1 (LTGF-β1), wherein the mature cytokine remains structurally sequestered and biologically inactive.

TGF-β1 is secreted through two distinct pathways: as an SLC associated with either GARP or LRRC33 or as a large latent complex (LLC) formed by binding to latent TGF-β-binding proteins (LTBPs), which subsequently anchor to the ECM via covalent interactions with ECM proteins such as fibronectin or fibrillin (18). The bioavailability and activity of TGF-β are tightly regulated by accessory molecules, collectively termed “TGF-β milieu molecules (19)”, including LTBPs (which anchor latent TGF-β to extracellular complexes), GARP (20)(which are expressed on Tregs, platelets, and endothelial cells (21–23)), and LRRC33 (which tethers TGF-β to the cell surface), as well as αvβ6 and αvβ8 integrins (which are critical for latent TGF-β activation). The activation of latent TGF-β1 is mediated by αvβ6 or αvβ8 integrin heterodimers, which bind the Arg-Gly-Asp (RGD) motif in LAP, generating mechanical tension that disrupts the constrained conformation and releases the active form (24).

**Figure 1:**
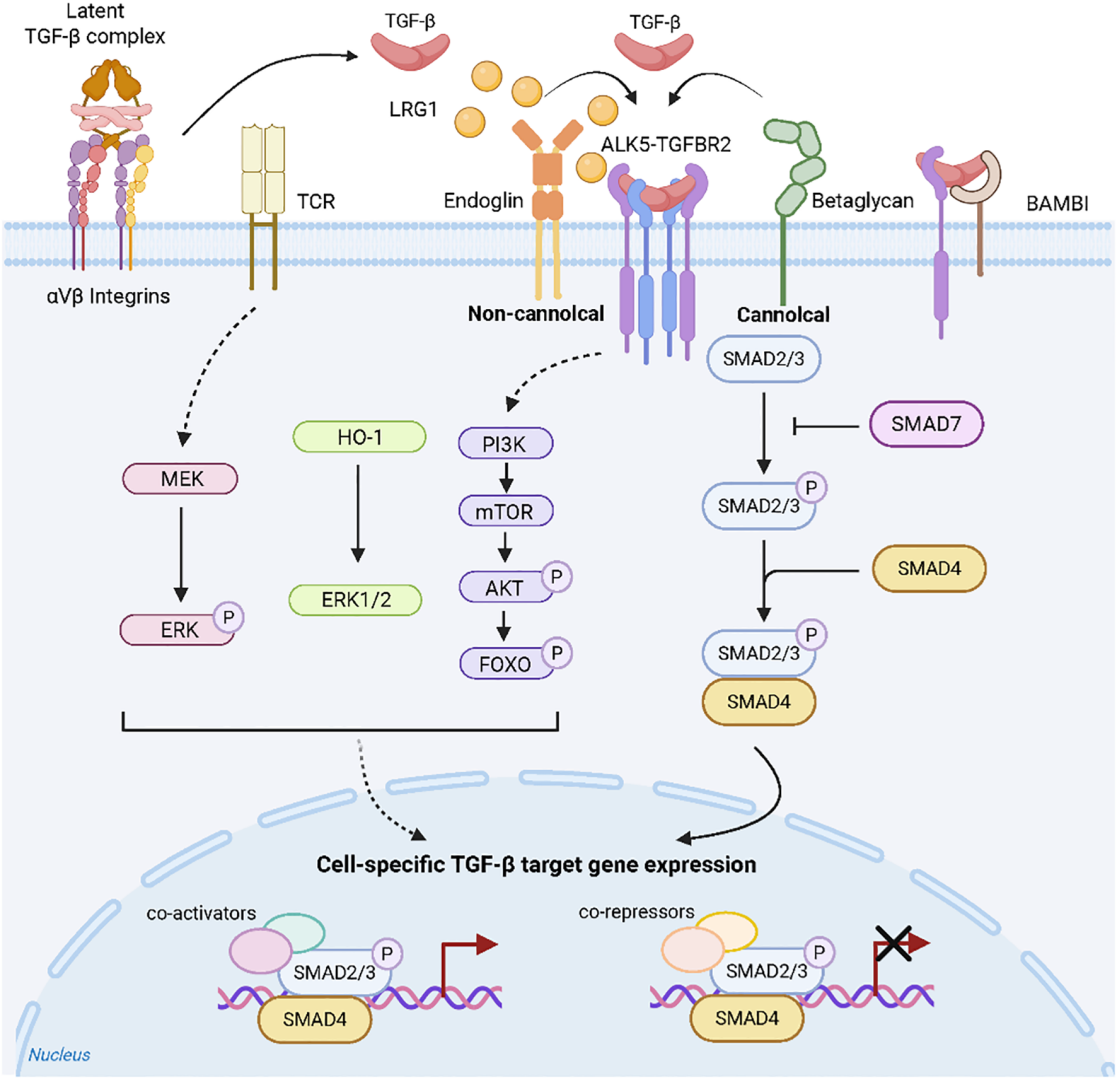
TGF-β signalling (25). The interaction of TGF-β with its specific receptors can initiate a series of intracellular pathways, which are categorized into Smad-dependent (canonical) and Smad-independent (non-canonical) signaling pathways, with their activation/transmission being finely regulated by various mechanisms.

TGF-β1 signalling pathway activation is initiated when the active ligand binds to TGF-β receptor type II (TGF-βRII) on target cells. This binding induces the phosphorylation and activation of TGF-β receptor type I (TGF-βRI). TGF-β1 signalling occurs through canonical (Smad-dependent) and noncanonical pathways (25). In the canonical pathway, activated TGF-βRI phosphorylates receptor-regulated Smads (R-Smads: Smad2 and Smad3), which then form a complex with Smad4. This complex translocates to the nucleus to regulate target gene transcription. Additionally, TGF-β1 utilizes multiple noncanonical pathways, including the PI3K/AKT/mTOR, MEK/ERKand ERK1/2, signalling cascades, to modulate diverse cellular functions (**Figure 1**) (25–29).

These non-canonical pathways regulate fibrotic processes either independently or cooperatively with Smad signaling, as evidenced by organ-specific models.Notably, in canine myxomatous mitral valve disease, TGF-β1 activates the PI3K/AKT pathway to drive myofibroblast transdifferentiation (25).Similarly, during bleomycin-induced pulmonary fibrosis, sinomenine concurrently suppresses both canonical (TGF-β1/Smad3) and non-canonical (PI3K/Akt) pathways to attenuate fibrogenesis (30).Furthermore, in murine non-alcoholic fatty liver disease, triptolide activates AMPK signaling, thereby inhibiting TGF-β1 expression, improving lipid metabolism, and reducing hepatic fibrosis (31).Critically, in radiation-induced intestinal fibrosis, TGF-β1 orchestrates connective tissue growth factor (CTGF) expression through dual activation of Smad-dependent signaling and the Rho/ROCK axis (32).

## 3 Research status of Smad2/3

### 3.1 Classification, structure and function of Smad family proteins

#### 3.1.1 Classification of Smad family proteins

The Smad protein family, comprising Mad proteins, Smad proteins, and their homologues (33, 34), serves as direct substrates for TGF-β family members. Although nine Smad proteins have been identified in total, only eight have been found in animals; these proteins can be functionally classified into three distinct subfamilies. First, receptor-regulated Smads (R-Smads), including Smad1, Smad2, Smad3, Smad5, and Smad8, are recognized by TβRI and function as downstream targets of TGF-β receptors. Notably, while Smad2 and Smad3 mediate TGF-β1 signalling, Smad1, Smad5, and Smad8 are phosphorylated by BMP receptors to transduce BMP - 7 signalling (35–37). Second, the common-mediator Smad (Co-Smad) subfamily consists solely of Smad4, which forms complexes with phosphorylated R-Smads in the cytoplasm before nuclear translocation to execute transcriptional regulation (38). Finally, the inhibitory Smads (I-Smads), comprising Smad6 and Smad7, function as negative regulators of the TGF-β signalling pathway (38).

#### 3.1.2 Structure and function of Smads family proteins

Smad family proteins, with molecular weights ranging from 42 – 60 kDa, exhibit high evolutionary conservation across species and typically consist of approximately 500 amino acids. Structurally, these proteins contain two conserved domains: an N-terminal MH1 (Mad homology domain-1) and a C-terminal MH2 (Mad homology domain-2), connected by a variable-length proline-rich linker region. Importantly, the MH1 domain has dual functions: it suppresses MH2 domain activity in the unactivated state, but upon activation, it binds specific DNA sequences to regulate transcription (39). Furthermore, comparative analysis revealed distinct conservation patterns between these domains: while the MH2 domain is highly conserved across all Smad family members, the MH1 domain is conserved only in R-Smads and Co-Smads and is completely absent in I-Smads (39–41).

Smad family proteins play essential roles in diverse cellular signalling pathways by regulating gene expression through two distinct mechanisms. First, they can directly modulate target gene transcription by either activating or repressing transcriptional activity (42). Alternatively, they may indirectly regulate gene expression by interacting with DNA-binding factors to control the expression of associated genes or other transcriptional regulators (43). For example, Smad3/4 complexes can form heteromeric complexes with E2F4/DP1 dimers that subsequently bind to the TGF-β inhibitory element (TIE) in the c-Myc promoter to repress target gene expression (44–46). Notably, while Smad2 and Smad3 serve as direct downstream effectors of TGF-β1 to promote tissue fibrosis, Smad7 functions as a negative regulator to suppress TGF-β1-mediated fibrotic responses (47).

### 3.2 Structural and functional similarities and differences between Smad2 and Smad3

Smad2 and Smad3 differ in many aspects. In the following sections, we compare the structure, activity, transcription level and function of Smad2 and Smad3.

#### 3.2.1 Similarities between Smad2 and Smad3

Smad2 and Smad3 exhibit significant structural and functional homology. Structurally, these proteins share 92% amino acid sequence identity in mice (48), with both containing conserved MH1 and MH2 domains connected by a proline-rich linker region. Functionally, as members of the R-Smad subfamily, they serve as downstream effectors of TGF-β1 signalling and demonstrate considerable functional redundancy (49, 50). Mechanistically, both proteins require TβRI-mediated phosphorylation before forming complexes with phosphorylated Smad4, a critical step for their subsequent nuclear translocation and transcriptional regulation (51–53). In terms of antioxidative stress, Smad2/3 can physically interact with Keap1 (Kelch-like ECH-associated protein 1) and its isoforms α and β through the EDGETSD and DLG motifs in the connection region between its MH1 and MH2 domains, mediating the expression of the Keap1–Nrf2 (nuclear factor E2-related factor 2, also called NFE2L2) pathway (54, 55).

#### 3.2.2 The activation and transcriptional differences of Smad2 and Smad3

Notably, Smad2 and Smad3 exhibit distinct activation patterns, primarily due to differences in their phosphorylation mechanisms. Although both proteins undergo phosphorylation, this process requires multiple regulatory proteins that have differential effects on each Smad isoform. Specifically, studies have revealed that SARA (Smad anchor for receptor activation) deficiency significantly impairs Smad2 phosphorylation but has a minimal effect on Smad3 (56, 57).

Smad2 and Smad3 modulate transcriptional activity both by repressing target gene promoters and through regulation by accessory proteins. Critically, their DNA-binding capacity depends on structural differences within the MH1 domain: Smad3 lacks the TID (transcriptional inhibitory domain) and binds DNA directly, whereas Smad2 requires Smad4 interaction for DNA association owing to its inhibitory TID (58, 59).

#### 3.2.3 Differences in function between Smad2 and Smad3

While both Smad2 and Smad3 are regulated by TGF-β1 signalling across multiple cell types, they exhibit significant functional divergence. Notably, during embryonic development, Smad2 expression is detectable at early stages, whereas Smad3 expression remains undetectable (60). Furthermore, genetic ablation studies have revealed differential impacts on viability: Smad2 knockout causes embryonic lethality, whereas Smad3-deficient mice survive postnatally but have markedly reduced lifespans (<6 months; minimum survival of 1 month) (61–63).

## 4 Cell regulatory function and mechanism of the TGF-β/Smad signalling pathway in fibrosis

### 4.1 Functions in cell proliferation and growth

Cellular proliferation, a fundamental biological process involving cell division, is distinct from cellular growth, which refers to an increase in cell size through protein synthesis, organelle biogenesis, and membrane expansion. Importantly, the TGF-β/Smad signalling pathway plays a critical role in negatively regulating both the proliferation and growth of normal cells. Mechanistically, the TGF-β-mediated inhibition of proliferation involves receptor activation of Smad2/Smad3, their subsequent complex formation with Smad4, their nuclear translocation, and the transcriptional repression of cell cycle-related genes. This regulatory mechanism is essential for maintaining tissue homeostasis and preventing aberrant proliferation. For example, in epithelial cells, the TGF-β–Smad axis suppresses proliferation by downregulating key cell cycle regulators (64). Furthermore, with respect to growth modulation, studies using human lung fibroblasts cultured in cystatin C (CST3)- and growth differentiation factor 15 (GDF15)-enriched media demonstrated that the inhibition of TGF-β/Smad signalling was correlated with reduced cellular proliferation (65).

### 4.2 Functions in cell differentiation and apoptosis

Importantly, the TGF-β/Smad signalling pathway critically regulates both cellular differentiation and apoptosis. Specifically, TGF-β promotes cell differentiation by activating Smad2/3 to modulate gene expression programs, as demonstrated by the psoralidin (PL)-induced differentiation of bone marrow mesenchymal stem cells into nucleus pulposus-like cells through this pathway (66). Conversely, during intervertebral disc degeneration, Grem1 accelerates disc cell apoptosis by inhibiting TGF-β-mediated Smad2/3 phosphorylation, thereby reducing inflammation-associated apoptotic events (67). In terms of apoptosis in myxoid mitral valve disease (MMVD), PI3K/AKT/mTOR signaling induced by TGF-β contributes to the pathogenesis of MMVD and plays a key role in the regulation of myofibroblast apoptosis (26).

### 4.3 Regulatory role in ECM

Fibrosis is characterized by the abnormal accumulation of extracellular matrix (ECM) components, such as collagen and fibronectin. The TGF-β signaling pathway plays a pivotal role in both physiological and pathological processes of tissue fibrosis (68). Mechanistically, TGF-β activation promotes the synthesis and deposition of ECM components—including collagens and fibronectin—through Smad2/3-mediated signalling (69). This regulatory function is particularly relevant to both tissue repair and fibrotic processes. Notably, in murine lung fibrosis models, ganoderic acid (GA) attenuates aberrant ECM accumulation by suppressing fibronectin expression and inhibiting hyperactivated TGF-β/Smad signalling (70). Conversely, during diabetic nephropathy, TGF-β drives pathological ECM deposition in glomerular mesangial cells via Smad-dependent pathways (71). Furthermore, activation of this pathway enhances ECM production and accelerates wound closure in diabetic foot ulcer mouse models (72). TGF-β1 can also be secreted in M2-type macrophages to promote myofibroblast proliferation, leading to ECM deposition (73). In the pathogenesis of idiopathic pulmonary fibrosis, the activation of TGF-β signaling pathway will accelerate the excessive production and deposition of ECM components, which will lead to the accumulation of fibrotic tissue. Functionally, the TGF-β/Smad signalling pathway serves as a multifunctional regulator of normal cellular physiology, maintaining tissue homeostasis through the precise coordination of proliferation, growth, differentiation, programmed cell death, and extracellular matrix turnover. However, dysregulation of this tightly controlled network frequently disrupts signalling equilibrium, thereby promoting pathological processes, including tumorigenesis and organ fibrosis. Notably, accumulating evidence reveals the dual regulatory nature of the pathway: it exerts tumour-suppressive effects during early carcinogenesis while paradoxically facilitating metastasis in advanced malignancies through mechanisms such as epithelial–mesenchymal transition promotion.

## 5 Regulatory role of the TGF-β/Smad signalling pathway in tissue fibrosis

Fibrosis represents a progressive pathological condition characterized by exaggerated wound-healing responses to persistent tissue injury across multiple organ systems. During this process, functional parenchymal cells are progressively replaced by excessive extracellular matrix deposition, ultimately compromising organ architecture and function. Notably, fibrogenesis affects diverse anatomical sites, such as cardiac and dermal tissues, beyond classical targets like lung, liver, and kidney. Critically, the TGF-β/Smad signalling pathway has been established as a central driver in numerous fibrotic disorders, where its aberrant activation correlates directly with increasing disease severity. Smad2 and Smad3, two primary downstream mediators of this pathway, exhibit high structural homology that confers partial functional redundancy, yet demonstrate distinct selectivity in their transcriptional targets (47, 74). Consequently, therapeutic strategies increasingly focus on targeting this pivotal pathway to attenuate pathological matrix accumulation.

### 5.1 Role of the TGF-β/Smad signalling pathway in hepatic fibrosis

The progression of hepatic fibrosis involves intricate interactions between hepatic stellate cells (HSCs) and various immune cells, particularly macrophages, within the liver. Extensive research has focused on elucidating the mechanisms underlying liver fibrosis, with particular emphasis on pharmacological interventions, signalling pathway modulation, and alterations in gene expression. Among these pathways, the TGF-β/Smad signalling pathway has been widely implicated in the pathological progression of liver fibrosis. Both *in vitro* and *in vivo* studies have demonstrated that astragaloside IV (75) and MOTS-c (mitochondrial open reading frame of the 12S rRNA type-c) (76) suppress HSCs activation and attenuate liver fibrosis by inhibiting the TGF-β1/Smad pathway through antioxidant stress mechanisms. Similarly, in CCL4-induced liver injury models, melatonin ameliorates hepatic fibrosis and improves liver function by downregulating TGF-β1/Smad signalling (77).

In nonalcoholic fatty liver disease (NAFLD), activation of the TGF-β1 signaling pathway leads to dysregulated hepatic lipid accumulation, thereby promoting the progression from a benign NAFLD condition to fibrosis, cirrhosis, and hepatic malignancies (78).Furthermore, (Pro)renin promotes HSCs activation and fibrosis in both human and murine HSCs, whereas its receptor knockdown TGF-β1/Smad3 pathway activity and mitigates fibrosis (79). Smad2 and Smad3 exhibit structural homology yet functional divergence. Multiple lines of evidence demonstrate that in HSCs, Smad2 overexpression downregulates collagen I and tissue inhibitor of metalloproteinase-1 (TIMP - 1) expression while augmenting matrix metalloproteinase-2 (MMP - 2) production. Conversely, Smad3 overexpression elicits diametrically opposed effects (80).Astaxanthin (ASTX) exerts antifibrotic effects by suppressing TGF-β1-induced mRNA and protein expression of α-smooth muscle actin (α-SMA) and procollagen alpha-1(I), thereby blocking TGF-β1 signal transduction and inhibiting Smad3 pathway activation in HSCs (81). Notably, the antifibrotic drug fluorofenidone (AKF-PD) inhibits HSCs autophagy via the TGF-β1/Smad pathway, thereby attenuating liver fibrosis (82). Moreover, studies on miRNAs have revealed that exosomal miR-342-3p derived from primary hepatic macrophages improves liver fibrosis by upregulating HPCAL1 (Hippocalcin-like protein 1) in HSCs, which subsequently inhibits TGF-β signalling (83).

### 5.2 Role of the TGF-β/Smad signalling pathway in renal fibrosis

Renal fibrosis, a hallmark of chronic kidney disease characterized by fibrotic alterations in the glomeruli and tubulointerstitium, frequently progresses to end-stage renal disease. Notably, dietary supplementation with eicosapentaenoic acid-enriched phospholipids (EPA-PLs) in spontaneously hypertensive rats suppresses TGF-β and Smad3 activation in renal tubular cells, enhances PI3K/AKT phosphorylation, diminishes the expression of proinflammatory cytokines (including IL - 1β and IL - 6), and consequently reduces tubulointerstitial fibrosis (84). Furthermore, research on ferroptosis in renal tubular epithelial cells (TECs) revealed a close link between its profibrotic mechanisms and the TGF-β/Smad pathway, and persistent ferroptosis effectively promotes fibrotic progression via this signalling axis (85). In experimental models of tubulointerstitial fibrosis, TGF-β1 ameliorates renal fibrotic progression through differential Smad-mediated regulation: Smad3 governs CTGF (connective tissue growth factor) and E-cadherin expression, while Smad2 modulates MMP - 2 expression. These findings demonstrate distinct target specificity of Smad2 versus Smad3 in tubulointerstitial fibrogenesis (86).

Moreover, therapeutic strategies targeting noncoding RNAs (such as miR-21, miR-145-5P, and miR-145-29b) have shown efficacy in mitigating nephritis and fibrosis in chronic kidney disease models, primarily through inhibition of the TGF-β1/Smad pathway (87). αKlotho, functioning as both an anti-fibrotic and anti-tumor agent, ameliorates impairment of renal structure and function in chronic kidney disease by inhibiting the binding of transforming growth factor-β (TGF-β) to its receptors (88). SIS3, a selective Smad3 inhibitor, directly suppresses Smad3-mediated collagen matrix expression and inhibits the accumulation of α-SMA-positive myofibroblasts in fibrotic kidneys. This blockade of endothelial-mesenchymal transition (EndMT) attenuates renal fibrosis and impedes disease progression (89). Lupus nephritis is a serious complication of systemic lupus erythematosus (SLE), driven by inflammation and fibrosis, which often leads to chronic kidney disease. Previous studies have shown that SPRY4-IT1 and TUG1 regulate TGF-β/Smad signaling to promote renal fibrosis in lupus nephritis (90). It is particularly important to note that the heart and kidneys are closely linked through the circulatory system. Primary dysfunction in one organ often leads to secondary dysfunction or injury in the other. These interactions shape cardiorenal syndrome, in which the activated fibrotic TGF-β1/Smad signaling pathway accelerates its pathological progression (91).

Macrophage subset differentiation is primarily categorized into classically activated M1 and alternatively activated M2 macrophages. In the kidney, persistent inflammation and prolonged release of factors like TGF-β lead to renal injury, ultimately resulting in renal fibrosis. Notably, studies have shown that macrophages are critically involved in renal fibrosis: within the kidney, M1 macrophages exert proinflammatory functions, and sustained inflammation coupled with the prolonged release of factors such as TGF-β drives progressive renal injury culminating in fibrosis. Conversely, M2 macrophages secrete TGF-β1 and fibroblast growth factor, promoting the proliferation of myofibroblasts and leading directly to ECM deposition (73). Similarly, studies on renal allograft rejection have demonstrated that METTL3 enhances the M2 macrophage-to-myofibroblast transition within this context by promoting the TGF-β1/Smad3 axis (92). Therefore, macrophages play a pivotal role in the pathogenesis and progression of renal fibrosis.

Type 2 diabetes (T2D) is a global health concern. Diabetic kidney disease in type 2 diabetes (T2DN) is one of the major microvascular complications of T2D and a leading cause of end-stage renal disease. TGF-β is a key regulator in renal fibrosis and mediates T2DN through its downstream Smad3-dependent mechanisms. Studies have demonstrated that treatment of prediabetic db/db mice (from 4 to 12 weeks of age) with SIS3, a Smad3 inhibitor, significantly reduced blood glucose levels and suppressed the elevation of serum creatinine, microalbuminuria, renal fibrosis, and inflammation, thereby substantially mitigating both T2D and T2DN. However, when db/db mice received late-stage SIS3 treatment during 8 – 16 weeks of age, although it inhibited the pathological progression of T2DN, it did not significantly improve T2D. This suggests that early intervention during the prediabetic stage may effectively prevent the development of both T2D and T2DN (93).

The signal transduction of TGF-β1 initiates upon its binding to the transforming growth factor beta type II receptor (TGFBR2), a constitutively active kinase (**Figure 1**). Subsequently, TGFBR2 phosphorylates and activates the type I receptor (TGFBRI), which then propagates downstream signaling through Smad2/3. Notably, the ubiquitin-proteasome system (UPS) serves as a critical mechanism for post-translational regulation. Of particular relevance, USP11— a key deubiquitinating enzyme—is significantly upregulated in fibrotic kidneys. Mechanistically, USP11 directly interacts with TGFBR2 and stabilizes it by counteracting its ubiquitin-mediated degradation via deubiquitination. Consequently, elevated TGFBR2 levels drive hyperactivation of the downstream Smad3/p53 pathway. This aberrant signaling cascade ultimately triggers renal tubular cell senescence and collectively exacerbates renal fibrosis progression (94).

Additionally, several investigations of natural compound extracts have revealed that their antifibrotic effects are mediated by the suppression of TGF-β/Smad signalling. For example, Ginkgo biloba leaf extract (EGb) ameliorates cisplatin-induced chronic renal interstitial fibrosis by downregulating the protein expression of TGF-β1, Smad2/3, and phosphorylated (p)-Smad2/3, thereby attenuating epithelial–mesenchymal transition in TECs (95). Similarly, ganoderic acid (GA) inhibits ECM deposition in the kidneys of unilateral ureteral obstruction (UUO) mice by suppressing fibronectin expression and preventing the overactivation of TGF-β/Smad signalling (69).

### 5.3 Role of the TGF-β/Smad signalling pathway in pulmonary fibrosis

Pulmonary fibrosis, a severe condition characterized by the replacement of normal lung architecture with excessive fibrous tissue, leads to significant impairment of lung function. Importantly, the TGF-β/Smad signalling pathway has been demonstrated to play a critical role in pulmonary fibrosis in diverse models, including those induced by radiation, perfluorooctanoic acid (PFOA), and bleomycin. Specifically, astilbin was shown to mitigate radiation-induced pulmonary fibrosis (RIPF) in both *in vitro* radiation injury models using murine lung epithelial cells (MLE - 12 and TC - 1) and *in vivo* RIPF models in C57BL/6J mice by inhibiting EMT through targeting the circPRKCE/TGF-β/Smad axis (96). Similarly, PFOA exposure induced pulmonary toxicity and fibrosis in adult male rats via activation of the TGF-β1/Smad signalling pathway (97). Furthermore, in bleomycin-induced mouse models, atractylodin (ATD) attenuated lung injury and fibrosis through the TGF-β/Smad pathway; concomitantly, ATD significantly suppressed both TGF-β1-induced EMT and fibroblast-to-myofibroblast transition *in vitro* (98). In the pathogenesis of pulmonary fibrosis, Forkhead box protein O3 (FOXO3) inhibits fibroblast activation and ECM deposition via its interaction with TGF-β1-induced Smad3, thereby attenuating idiopathic pulmonary fibrosis (IPF) (93, 99). Moreover, research into miRNAs underscores the importance of this pathway: for example, miR-326-mediated overexpression of nuclear factor I-B (NFIB) inhibits TGF-β-induced EMT and reverses pulmonary fibrosis (100), while miR-486-5p alleviates fibrosis in radiation-induced lung injury (RILI) by suppressing Smad2 and activating Akt (101).

### 5.4 Role of the TGF-β/Smad signalling pathway in cardiac fibrosis

Myocardial fibrosis, characterized by the excessive accumulation of ECM synthesized by cardiac fibroblasts (CFs), represents a common pathophysiological process in various cardiac diseases, including myocardial infarction (MI), hypertensive heart disease, and cardiomyopathies. Crucially, this pathological remodelling has detrimental effects on cardiac function. Two distinct types of myocardial fibrosis exist: reactive fibrosis and reparative (replacement) fibrosis. Importantly, the TGF-β1 signalling pathway is crucially involved in myocardial fibrosis and significantly influences disease progression and severity. In the context of MI, activation of the TGF-β/Smad signaling pathway can exert protective effects. For instance, in cardiac fibroblasts (CFs), overexpression of Carbonic Anhydrase III (CAIII) potentiates fibroblast activation via the Smad7-TGF-β/Smad2/3 signaling axis. Subsequent activation of these fibroblasts promotes cardiac wound healing (102). In contrast, the ubiquitin-like protein HLA-F adjacent transcript 10 (FAT10) mediates cardiac fibrosis through Smad3 in MI (103). Pulmonary arterial hypertension (PAH) is characterized by excessive proliferation and anti-apoptosis of pulmonary artery smooth muscle cells (PASMCs). Studies have shown that DJ - 1 alleviates DJ - 1-induced PASMCs injury by inhibiting TGFβ/Smad signaling pathway (104).

Mounting evidence shows that the TGF-β1 signalling pathway is a pivotal therapeutic target for cardiac fibrosis. For example, in the context of atrial fibrillation (AF), inhibition of the TGF-β1/Smad pathway mitigates angiotensin II (Ang II)-mediated fibrotic remodelling (105). Furthermore, studies utilizing human AF samples, TGF-β-treated human atrial endocardial endothelial cells (AEECs), and cardiac-specific TGF-β transgenic mice have demonstrated that miR-181b ameliorates AF fibrosis by suppressing the TGF-β-induced endothelial-to-mesenchymal transition (EndMT) (106). Similarly, research in human cardiac fibroblasts (HCFs) revealed that miR-452-5p regulates fibrotic progression under SCN5A deficiency by targeting the TGF-β/Smad axis (107). Moreover, investigations in diabetic cardiomyopathy (DCM) models have shown that the loss of natriuretic peptide receptor C (NPRC) in both DCM mice and patient myocardia upregulates TGF-β-induced factor homeobox 1 (TGIF1); consequently, TGIF1 inhibits Smad2/3 phosphorylation, thereby attenuating cardiac fibrosis and improving remodelling and function in diabetic mice (6).

Notably, the ubiquitin–proteasome system (UPS) plays a significant role in the development of myocardial fibrosis by regulating the degradation and synthesis of proteins involved in both the TGF-β-dependent and the TGF-β-independent profibrotic pathways. Specifically, members of the USP family can modulate this process by targeting signalling molecules and transcription factors critical for fibroblast proliferation and differentiation. For example, USP10 promotes fibrosis through the TGF-β/Smad signalling pathway by deubiquitinating Smad4; similarly (108), USP15 facilitates fibroblast activation and ECM production by activating the TGF-β/Smad pathway (109).

### 5.5 Role of the TGF-β/Smad signalling pathway in skin fibrosis

Skin fibrosis, characterized by excessive deposition and abnormal proliferation of the ECM, underlies various pathological conditions, including oral submucous fibrosis, scleroderma (systemic sclerosis, SSc), and scarring. Aberrant scar formation, including keloids and hypertrophic scars, is associated with a pathological, dysregulated chronic inflammatory wound healing process. The TGF-β/Smad signaling pathway represents the classic pathway regulating collagen synthesis in fibroblasts and myofibroblasts (78). Studies demonstrate that Zyxin effectively attenuates keloid formation through inhibitory regulation of the TGF-β signaling pathway (110). In support of this notion, osthole attenuates myofibroblast activity in oral submucous fibrosis by inhibiting the TGF-β/Smad2 pathway (111). Moreover, in a bleomycin (BLM)-induced SSc skin fibrosis mouse model generated by repeated subcutaneous injections, both adipose-derived mesenchymal stem cells (AMSCs) and their exosomes reduced dermal thickness and the collagen volume fraction, concurrently suppressing α-smooth muscle actin (α-SMA) and type III collagen (COL3A1) expression in skin tissue via the TGF-β1/Smad3 axis (112). Similarly, artesunate effectively mitigated hypertrophic scar formation in a rabbit ear model by inhibiting endothelial–mesenchymal transition and fibroblast activation through the downregulation of key proteins in the TGF-β/Smad signalling pathway (113).

### 5.6 Role of the TGF-β/Smad signalling pathway in fibrosis in other diseases

Fibrotic diseases are pathological processes affecting multiple organ systems. In addition to well-characterized fibrosis in organs such as the liver, heart, skin, and kidneys, other tissues, including the eyes and mammary glands, are also susceptible. For example, in a streptozotocin (STZ)-induced diabetic Sprague–Dawley (SD) rat model, Poldip2 overexpression contributes to diabetic retinal fibrosis via the TGF-β1/Smad signalling pathway (114). In contrast, in mouse corneal mechanical injury models, calcitonin gene-related peptide (CGRP) reduces TGF-β1 signalling and prevents TGF-β1-mediated stromal fibroblast activation and tissue fibrosis (115). Similarly, in triple-negative breast cancer (TNBC), PCK2 modulates Smad3 expression and phosphorylation by inhibiting TRIM67-mediated Smad3 ubiquitination; this action consequently enhances TGF-β-stimulated Smad3 activity and activates TGF-β/Smad3 signalling (116). Neuroblastoma (NB) is a cancer arising from neuroblasts. In the context of NB treatment, atypical protein kinase Cs (aPKCs) upregulate the Akt1/NF-κB and TGF-β pathways by binding to 14 - 3–3 and Smad2/3 (117). Regarding juvenile polyposis syndrome (JPS), a rare autosomal dominant disorder, genetic testing reveals aberrant Smad4 expression, which serves as a biomarker for rapid diagnosis of this disease (118). In order to facilitate an intuitive understanding of the main contents of Part 5, statistics are presented in the form of tables (**Table 1**).

**Table 1:** Regulatory roles of TGF-β/Smad signaling pathway in organ fibrosis.

Organ	Fibrosis type	Core mechanism	Key interventions/findings	Models	References
Liver	Hepatic fibrosis	TGF-β/Smad drives HSCs activation & ECM deposition	• Astragaloside IV & MOTS-c: Inhibit TGF-β1/Smad via antioxidant stress • Melatonin: Downregulates TGF-β1/Smad in CCl_4_ models • AKF-PD: Inhibits HSC autophagy via TGF-β1/Smad • miR-342-3p: Inhibits TGF-β via HPCAL1	*In vitro* HSCs; CCl_4_/NAFLD/BLM models; Mice	(74–80)
Kidney	Renal fibrosis (CKD, T2DN)	TGF-β/Smad3 mediates tubulointerstitial fibrosis & inflammation	• EPA-PLs: Suppress TGF-β/Smad3 & inflammation • Anti-Smad3 therapy: Reduces T2D/T2DN severity in db/db mice • αKlotho: Blocks TGF-β receptor binding • Ginkgo biloba/Ganoderic acid: Downregulate TGF-β1/Smad2/3	Spontaneously hypertensive rats; db/db mice; UUO models	(73, 81–89)
Lung	Pulmonary fibrosis (IPF, RIPF)	TGF-β1-induced Smad3 promotes fibroblast activation & ECM deposition	• Astilbin-salvianolic acid B: Targets circPRKCE/TGF-β/Smad • FOXO3: Inhibits fibrosis via Smad3 interaction • miR-326: Inhibits EMT via NFIB • miR-486-5p: Suppresses Smad2	Radiation/PFOA/bleomycin models; Mice	(90–95)
Heart	Myocardial fibrosis (Reactive/Reparative)	TGF-β1/Smad3 axis mediates ECM overproduction by CFs	• CAIII overexpression: Activates Smad7-TGF-β/Smad2/3, leading to wound healing • FAT10 deletion: Reduces Smad3-mediated fibrosis • miR-181b/miR-452-5p: Suppress EndMT/fibrosis via TGF-β/Smad	MI models; Human AF samples; Diabetic mice	(6, 96–103)
Skin	Cutaneous fibrosis	TGF-β/Smad regulates collagen synthesis in fibroblasts/myofibroblasts	• Zyxin: Attenuates keloids via TGF-β inhibition • Osthole: Inhibits TGF-β/Smad2 in oral fibrosis • AMSCs/Exosomes: Suppress α-SMA/COL3A1 via TGF-β1/Smad3 • Artesunate: Downregulates TGF-β/Smad in scars	BLM-induced SSc models; Rabbit ear scars; Human tissue	(78, 104–107)
Other	• Diabetic retinal fibrosis • TNBC desmoplasia • NB progression • JPS diagnosis	TGF-β/Smad drives ECM pathology in non-canonical organs	• Poldip2: Promotes retinal fibrosis via TGF-β1/Smad • PCK2: Enhances TGF-β/Smad3 in TNBC • aPKCs: Upregulate TGF-β via Smad2/3 in NB • Smad4: Biomarker for JPS	STZ-diabetic rats; TNBC cells; Mouse models; Human genetics	(108–112)

## 6 Discussion

**Figure 2:**
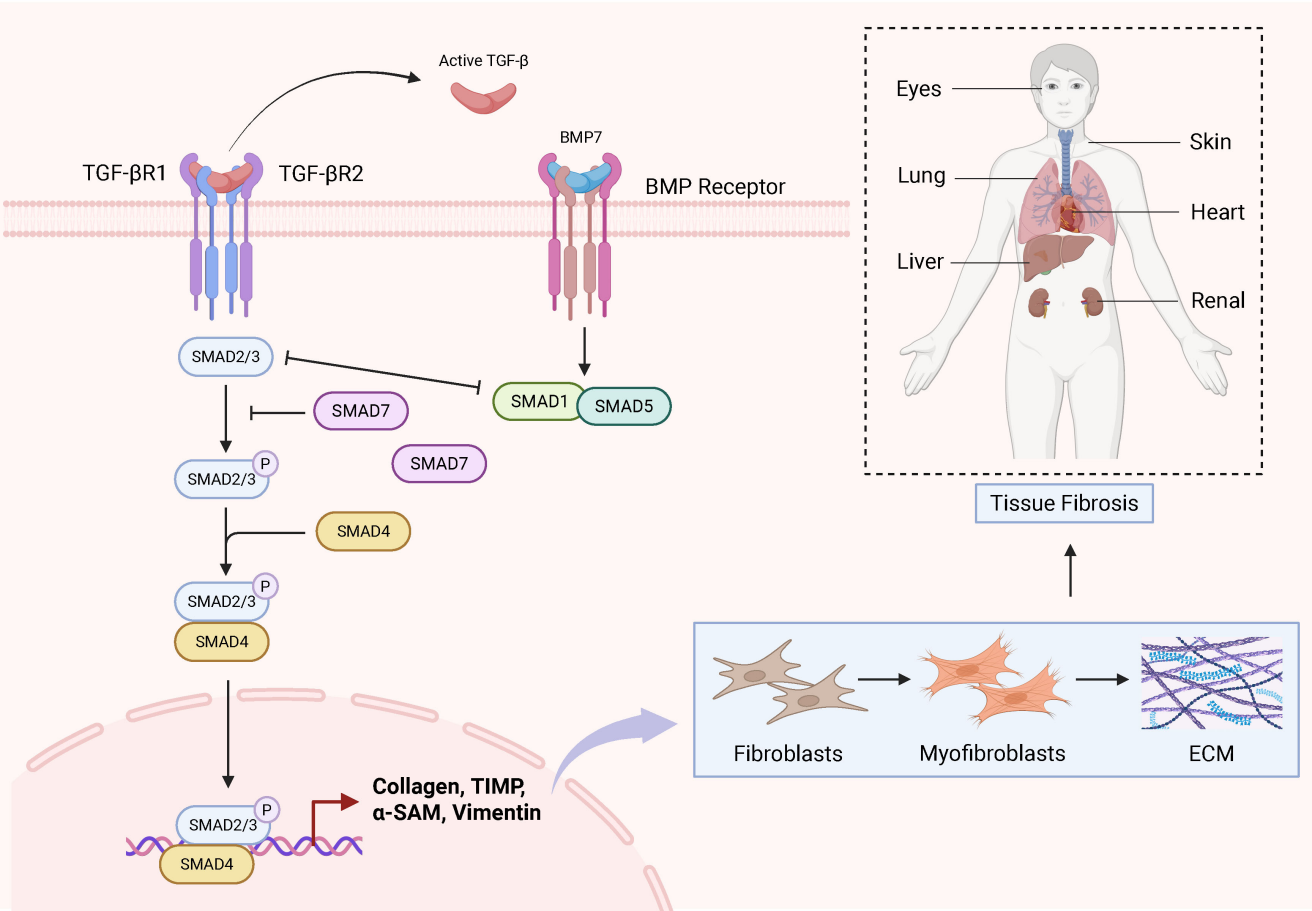
Schematic diagram of the TGF-β/Smad signalling pathway in fibrosis in various tissues.

Fibrotic diseases involve complex pathogenic mechanisms that frequently progress to organ failure, yet effective therapeutic interventions remain limited. Therefore, this review aims to elucidate the molecular mechanisms through which the TGF-β1/Smad signaling pathway drives fibrotic pathogenesis. Specifically, we focus on its core biological functions: (1) driving pathological cellular phenotypic transitions, (2) promoting excessive extracellular matrix (ECM) deposition, and (3) suppressing ECM degradation—collectively culminating in structural disruption and functional decline of affected organs. Ultimately, this work establishes a foundation for developing targeted therapeutic or prophylactic agents against this pivotal pathway. Importantly, numerous recent studies have focused on elucidating the pivotal role of the TGF-β/Smad signalling pathway in these disorders. Specifically, this pathway has been established as a key regulatory mechanism in multiple fibrotic conditions, including hepatic, pulmonary, cardiac, and renal fibrosis (**Figure 2**). Furthermore, oxidative stress and autophagy are closely involved in fibrosis pathogenesis. For example, The Keap1-Nrf2 pathway represents a canonical antioxidant response mechanism. Keap1 interacts with Smad2/3 proteins, and upon Nrf2-mediated antioxidant activation, TGF-β/Smad signaling is downregulated (54, 119). In experimental models of diabetic nephropathy, Mefunidone (MFD) treatment reduces ROS generation, suppresses TGF-β1/Smad pathway activation, and inhibits epithelial-mesenchymal transition (EMT) (120). Though the role of autophagy in fibrosis appears to be context-dependent, with both pro- and anti-fibrotic effects reported. Additionally, other signalling pathways contribute to fibrotic pathogenesis. Notably, the AMPKα/MMP9 axis attenuates skeletal muscle fibrosis, while PPAR-γ/NLRP3/NF-κB signaling mitigates pulmonary fibrosis progression (121, 122). Collectively, this evidence suggests that, therapeutic strategies targeting a single pathway may be insufficient, necessitating a comprehensive approach that considers the interplay of multiple signalling networks.

In the future, promising research directions include first, further delineating the precise regulatory mechanisms of the TGF-β/Smad pathway to identify novel molecular targets; second, investigating its interactions with other pathways, such as those involving oxidative stress, to construct a more holistic map of fibrotic mechanisms (76); third, developing innovative therapeutic modalities, including extracellular vesicles (exosomes), targeted therapies, and phytomedicine formulations, aiming to enhance efficacy and minimize adverse effects (123–125); and finally, elucidating how environmental determinants (e.g., pollutants) influence fibrotic initiation/progression to inform preventive strategies (126, 127). Collectively, these approaches will advance our rapidly evolving understanding of fibrotic pathogenesis and accelerate the translation of novel interventions towards improved patient outcomes, while simultaneously presenting challenges in drug development—particularly regarding tissue specificity and safety profiles.

## 7 Conclusion

The TGF-β/Smad pathway is the master regulator of multi-organ fibrosis, driving pathological ECM accumulation via fibroblast activation, EMT, and suppressed matrix degradation. Key organ-specific mechanisms include: hepatic stellate cell activation (e.g.,inhibited by astragaloside IV/miR-342-3p); Smad3-mediated renal inflammation (e.g., attenuated by SIS3/αKlotho); TGF-β1-induced pulmonary fibroblast transition (e.g., targeted by miR-326/FOXO3); USP-dependent cardiac Smad deubiquitination; and dermal collagen overproduction (e.g., suppressed by osthole/exosomes). Therapeutic inhibition consistently attenuates fibrosis across models, validating its clinical potential. Future research must resolve tissue-specific regulatory divergence (e.g., Smad2 vs. Smad3) and develop combinatorial strategies (e.g., miRNA-phytomedicine hybrids) for precision antifibrotic therapy.
